# High presacral collection approached through the perineal route: A novel computed tomography-guided technique

**DOI:** 10.4102/sajr.v25i1.2014

**Published:** 2021-03-11

**Authors:** Shuchi Bhatt, Harshit Bansal, Sagar Nayak, Saumya Dangwal

**Affiliations:** 1 Department of Radiodiagnosis, Faculty of Health Sciences, University College of Medical Sciences and GTB Hospital, Dilshad Garden, Delhi, India; 2 Department of Orthopaedics, Faculty of Health Sciences, University College of Medical Sciences and GTB Hospital, Dilshad Garden, Delhi, India

**Keywords:** MDR, TB, infectious medicine, interventional radiology, trans-perineal

## Abstract

For a definitive diagnosis of abdomino-pelvic lesions, percutaneous aspiration or biopsy is often necessary; however, finding a safe ‘window’ for access is challenging. This case report discusses a novel method to approach a deep pelvic collection and also briefly reviews the various approaches to access such lesions. A sample was obtained from a non-resolving presacral collection using a CT-guided percutaneous, trans-perineal approach with repeated sessions of hydro-dissection. Successful aspiration and analysis revealed multi-drug resistant tuberculosis, thus guiding appropriate management.

## Introduction

Imaging diagnosis of presacral and deep pelvic lesions is usually non-specific and histo-pathological analysis is necessary for establishing the diagnosis. Image-guided percutaneous aspiration or biopsy is a safe and effective procedure. However, deep pelvic lesions pose a challenge for interventional radiologists as the overlying bowel, bladder, vessels, bones and uterus, and adnexa in female patients often preclude safe access.^1^ Organ displacement techniques, such as hydro-dissection, may provide ‘windows’ to safely access lesions that are blocked by overlying structures.^2,3^

Fine needle aspiration is sufficient for evaluating infected collections and usually requires a small calibre needle. However, chronic collections (especially tubercular) are frequently dense and organised, requiring a large-bore needle (18G or more), thus precluding transgression of the bowel or bladder to approach deep-seated pelvic lesions. There is also a risk of fistula formation and spread of infection in cases of tubercular aetiology. In addition to establishing the diagnosis of tuberculosis, percutaneous aspiration may be required for drug sensitivity testing for multi-drug resistant tuberculosis (MDR-TB) in cases of inadequate or poor response to anti-tubercular treatment. A safe access route is, therefore, required in order to obtain the specimen and to prevent inadvertent complications, which can be achieved by scrupulous planning under image guidance.

The success, effectiveness and safety of percutaneous pelvic needle aspiration or biopsy are dependent on a thorough understanding of the cross-sectional anatomy of the pelvis.^4,5,6,7,8,9,10^ Both sonography and computed tomographic (CT) allow direct simultaneous visualisation of the pelvic structures and pathology. Although relatively low-lying lesions in the pelvis may be approached transrectally, using ultrasound guidance makes it difficult to approach more cephalad lesions in the presacral space.

Several percutaneous approaches have been described for overcoming various challenges in accessing pelvic and/or perineal lesions under CT guidance. A radiologist should be aware of the technical and patient-related limitations to avoid injury of the vital intervening structures whilst approaching the lesion.

A novel CT-guided technique specifically tailored to suit a difficult approach to a high presacral collection is discussed in detail. The challenges presented and the solutions devised to overcome them are detailed in this case report. Successful aspiration of the collection avoided surgery, helped obtain the tissue sample and also permitted appropriate treatment to be instituted.

## Case presentation and procedure

A 38-year female patient with Pott’s spine involving the L5-S1 vertebrae showed no significant clinical or imaging improvement after 15 months of anti-tubercular treatment. The possibility of MDR-TB or an alternative aetiology was considered. Magnetic resonance imaging (MRI) of the spine revealed a well-defined destructive lytic lesion involving the L5 and S1 vertebral bodies, along with a large presacral soft tissue component with internal calcifications. Comparison with previous MR imaging indicated an increase in the collection size and associated-bone destruction. Histo-pathological evaluation was deemed necessary and the patient was referred to the radiology department for image-guided aspiration.

Anatomy of the pelvic structures and standard percutaneous CT-guided approaches to detect deep pelvic lesions were carefully reviewed. Use of the classic anterior or lateral trans-abdominal approach would require transgression of interposed bowel loops or a distended urinary bladder and was therefore considered inappropriate. A trans-gluteal approach was not considered, as the collection was located far above the greater sciatic foramen. An antero-lateral extra-peritoneal approach was rejected because of the location of the internal iliac neurovascular bundle along the proposed needle path. A trans-osseous approach through the sacrum was also not possible as the collection was anterior to the sacral promontory. A para-coccygeal-infragluteal approach was deemed impossible because of the high position of the collection.

The high position of the presacral collection (mean HU 35) at the S1–2 level made conventional percutaneous techniques practically impossible to use. The greatest challenge was to negotiate the sacral curve to reach the collection without injuring the important structures. We, therefore, resolved to collect the sample using a novel trans-perineal approach. The distance of the collection from the expected site of needle entry into the perineum was approximately 9 cm and thus, required a large-bore needle of long length. However, as there was the potential to transgress the anal canal and rectum using this approach, another prerequisite was to displace the rectum off the sacro-coccygeal curve so as to provide a safe needle pathway.

**FIGURE 1:**
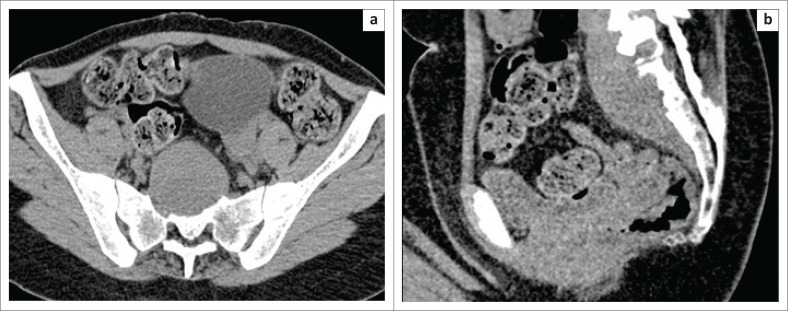
(a and b) Axial and sagittal images at the level of sacrum demonstrate an elongated hypodense collection in the high presacral region resulting in erosion of the anterior aspect of the upper sacral vertebrae. The collection was unapproachable anteriorly because of the presence of bladder and bowel loops and is located in close proximity to the rectum antero-inferiorly.

The patient was counselled about the procedure and written informed consent was obtained. The patient was positioned in the left lateral knee-chest position on the gantry table and a non-contrast computed tomography (NCCT) scan was obtained from the level of the L5 vertebra to the inferior limit of the gluteal region. The 3D sections were reconstructed and images analysed again for the proposed needle pathway (Figure 1). A review of the sagittal sections showed the rectum lying in the needle pathway and thus required anterior displacement in order to avoid injury. Hydro-dissection seemed to be a viable option for achieving this.

**FIGURE 2:**
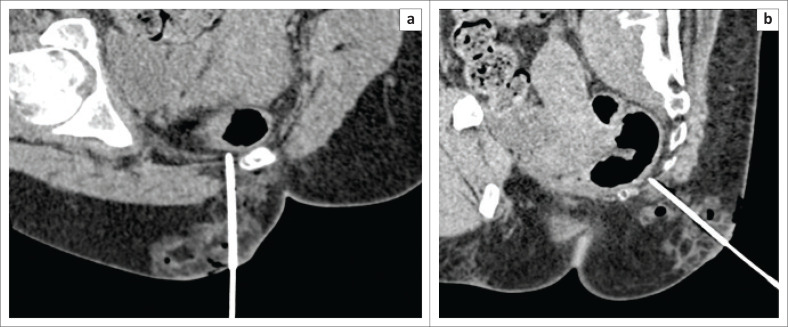
(a and b) Oblique axial and sagittal images show the tip of the needle (21G) at the level of the S5 vertebra in the presacral space with the rectum immediately antero-lateral to it.

**FIGURE 3:**
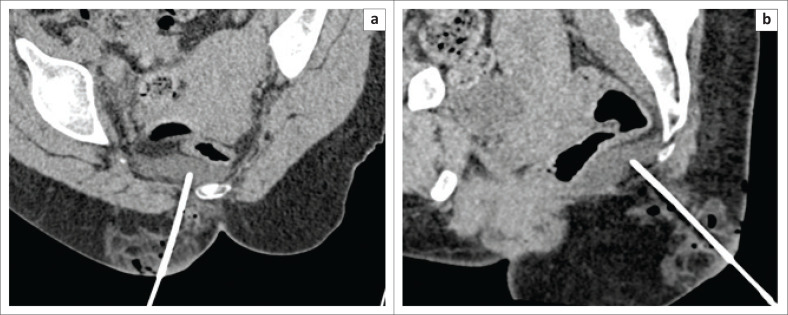
(a and b) Oblique axial and sagittal images after instillation of 0.9% saline showing anterior displacement of the rectum and widening of the presacral space.

The initial aim was to reach the presacral space at the S5 level. The tip of the coccyx was palpated and a point 3 cm lateral and 2 cm anterior to it was selected on the right buttock and marked. After cleaning and draping, 10 mL of local anaesthesia was infiltrated, and a 21G spinal needle was inserted and slowly advanced anteriorly, cranially and medially at an angle of 30 degrees along the lateral sacro-coccygeal border until the tip of the needle reached the retro-rectal space at the S5 level (Figure 2). After confirming the position of the needle tip on limited axial cuts, 20 mL of 0.9% saline was instilled so as to displace the rectum anteriorly (Figure 3).

**FIGURE 4:**
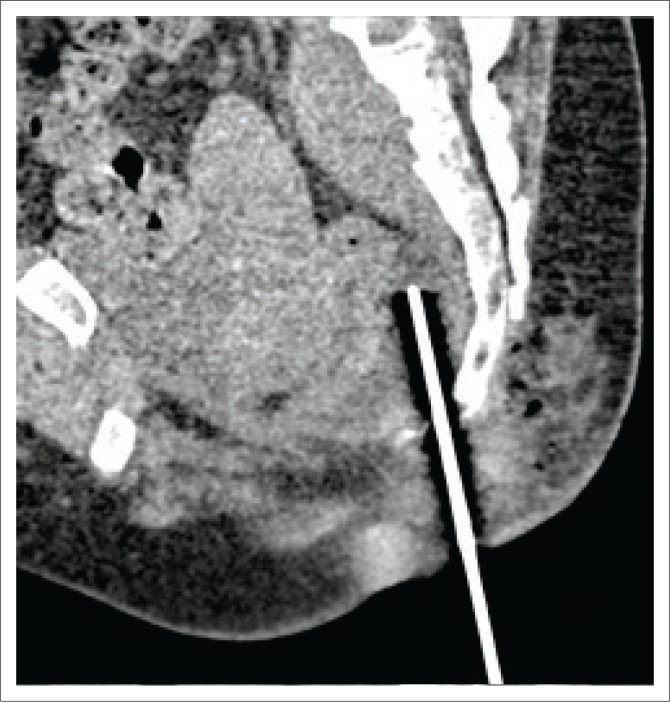
Sagittal image showing the progressive cranial advancement of the needle in the presacral space, made possible by anterior displacement of the rectum through repeated hydro-dissection. The needle is approximating the caudal extent of the collection.

The spinal needle was then withdrawn and then re-inserted from a para-median point (to avoid injury to the midline ano-coccygeal ligament) to the same vertical level after infiltration with local anaesthetic. The needle was carefully advanced cranially and hydro-dissection was repeated with 20 mL saline to further displace the rectum anteriorly. This was repeated until the needle could reach the target point (Figure 4).

**FIGURE 5:**
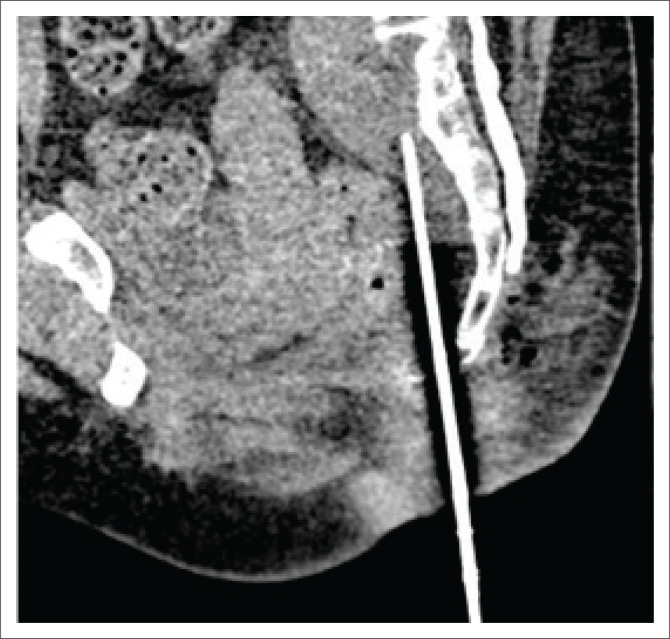
Oblique sagittal multiplanar reconstruction image indicating the 18G co-axial needle advanced cranially into the collection. Upon aspiration, thick viscous content was obtained which was sent for histo-pathological analysis in a sterile container.

As the collection was viscous and deep, an 18 G co-axial needle was chosen and was introduced utilising the spinal needle as a guide. Upon confirmation of accurate needle placement, the spinal needle was removed and the co-axial system was further advanced cranially into the collection (Figure 5). Upon removal of the stylet, 4 mL – 5 mL of thick purulent material was aspirated, placed into sterile containers and sent for histo-pathological confirmation and a cartridge-based nucleic acid amplification test (CB-NAAT).

Once the procedure was completed, the needle was withdrawn and the puncture site was sealed with tincture iodine. No procedural or post-procedural complications were encountered.

Analysis of the sample revealed MDR-TB, resistant to both isoniazid and rifampicin.

## Discussion

Image-guided percutaneous aspiration is a widely accepted and commonly performed minimally invasive procedure that provides a means of procuring tissue samples for a variety of diagnostic purposes.^2^ Ultrasound-guided percutaneous fine needle aspiration is often considered to be the simplest and most efficient technique. However, CT-guided puncture has increased precision as it provides greater spatial resolution and is helpful in cases where US-guided puncture is not possible because of superimposed intestinal gas, bone or adipose tissue.^7^ Various imaging guided approaches have been described in the literature.

### Classic anterior or lateral trans-abdominal approach

This approach is the most common. During this technique, the needle is inserted through the lower abdominal wall and peritoneum with the patient in the supine or lateral decubitus position, avoiding the inferior epigastric vessels, deep circumflex iliac vessels and bowel. However, if bowel transgression with a thin 22G needle occurs, this is considered safe. Emptying the bladder allows access to deeper lesions.

This technique is best suited for lesions cranial, anterior or lateral to the bladder. Ultrasonography of a full bladder is required to visualise lesions behind it; the echoes traverse the bladder to reach the target. However, interventionally, peritoneal or urinary bladder puncture is painful and the presence of surgical dressings or colostomy bags may preclude access in post-operative patients.

### Trans-gluteal or trans-sciatic approaches

This method places the patient in the prone position and is used for posterior pelvic lesions located in the lower pelvis that are not safely accessible because of intervening bowel, bladder, uterus and iliac vessels. The needle trajectory is through the sacro-spinous ligament via the caudal part of the greater sciatic notch below the level of pyriformis and as close as possible to the edge of the sacrum in order to avoid damaging the neurovascular structures exiting the pelvis. The main drawback is the risk of injury to the sciatic nerve, gluteal vessels and sacral plexus.

### Antero-lateral extra-peritoneal approach

The technique involves needle transgression through the ilio-psoas muscle in the supine position. It is suited for lesions located along the medial aspect of the iliopsoas muscle, usually pelvic lymph nodes. Masses or loculated fluid along the pelvic sidewall and adnexal masses located posterior to the vessels can also be sampled. Contrast material administration is occasionally necessary with CT guidance in order to define the vascular structures. The major risk involves injury to iliac vessels, the femoral nerve and ureters.

### Trans-osseous approach

This procedure can be attained via the trans-sacral or trans-iliac routes. During the trans-sacral approach, the needle should be advanced through the medial (between the central canal and foramina) or extreme lateral part of the sacrum. In a trans-iliac approach, the needle is advanced through the narrow part of the iliac wing taking care to avoid injury to the ureters and gonadal vessels. This technique specifically targets presacral and posterior pelvic lesions not accessible by the trans-gluteal approach because of the location above the greater sciatic foramen or interposition of vascular structures. Besides being painful, there is a risk of injuring the sacral nerves.

Needle stabilisation is better with the trans-gluteal, antero-lateral extra-peritoneal or trans-osseous approaches as compared to the trans-abdominal approach.

### Trans-perineal, para-coccygeal or infra-gluteal approach

This strategy has also been described for deep pelvic lesions located caudally in the presacral and ischio-rectal spaces. The coccyx is used as a palpable bony landmark for choosing the site of needle entry under CT guidance. Most limitations of the trans-gluteal approach are overcome by this technique, other than minimal patient discomfort. The gluteal muscles and sciatic plexus are safely avoided. However, the insertion of the needle is considered more difficult as compared to the trans-gluteal approach.^11^ The close approximation of the rectum in the sacral concavity limits this approach for caudally placed presacral lesions. In our case, however, we approached a high presacral collection by modifying this technique at various stages.

The trans-vaginal and trans-rectal approaches have also been described for drainage of collections lying in close proximity to the vaginal vault and in the ischio-rectal spaces.

Various non-invasive methods have been described to achieve safe needle trajectories for percutaneous puncture by adjusting angulation of the CT gantry, patient positioning, timing of respirations with needle passage and use of compression devices.^3,12,13,14^ Several authors have also described various methods for physically displacing vital structures away from the target lesion to create percutaneous access routes using agents such as sterile water, 0.9% normal saline solution, 5% dextrose in water and carbon dioxide.^12,13,14^

Although large presacral lesions (or smaller ones that are in the anterior and caudal aspect of the presacral space) may be approached via the greater sciatic foramen,^4^ adjacent to the coccyx^11^ or transrectally,^15^ a high, small lesion close to the anterior surface of the sacrum is a more difficult target. In our patient, we were able to avoid invasive surgical intervention by successfully using an innovative CT-guided technique to reach a high presacral collection for obtaining tissue samples for re-confirmation of the aetiology and detecting the reason for non-response to anti-tuberculous treatment.

## Conclusion

This case report discusses the various percutaneous image-guided techniques utilised for accessing deep pelvic lesions or collections with a focus on a novel technique used to approach a presacral collection through the percutaneous, trans-perineal, para-coccygeal approach. Additionally, we adjusted the patient’s position and utilised hydro-dissection to achieve anterior displacement of the rectum and create a safe needle pathway in the sacral curve, making this case unique.

Familiarity with the cross-sectional pelvic anatomy and various percutaneous approaches facilitates planning of a safe access route to deep-seated pelvic/presacral lesions and helps avoid injury to major neurovascular structures and other viscera. Knowledge of the advantages and disadvantages of each technique allows the radiologist to choose the optimal approach in a given setting.^1^ Displacement of important structures by injecting saline can be achieved to create access routes for image-guided percutaneous abdominal or pelvic biopsies/ drainages of lesions that are otherwise inaccessible because of their depth or proximity to vital structures.

Tubercular collections, in particular, can create specific challenges, such as bowel, bladder and peritoneal transgression, which are not advisable because of the risk of persistent fistula formation and spread of infection. Furthermore, the viscous nature of the collections limits the size of needle used for aspiration. As evident in this case, a tailor-made approach was deemed necessary, considering the location of the lesion and specific challenges posed.
